# Age-related hepatic tolerance to ischemia-reperfusion injury in a rat model of extended hepatectomy

**DOI:** 10.3389/fsurg.2026.1902759

**Published:** 2026-10-09

**Authors:** Dmitry G. Akhaladze, Ivan V. Tverdov, Alexander A. Andreev-Andrievskiy, Mikhail A. Mashkin, Olga Yu. Frolova, Nikolay S. Grachev

**Affiliations:** 1 Department of Pediatric Surgery and Urology-Andrology named after Prof. L.P. Alexandrov, I.M. Sechenov First Moscow State Medical University of the Ministry of Health of the Russian Federation (Sechenov University), Moscow, Russia; 2 Dmitry Rogachev National Medical Research Center of Pediatric Hematology, Oncology and Immunology, Ministry of Health of the Russian Federation, Moscow, Russia; 3 Institute for Biomedical Problems of the Russian Academy of Sciences, Moscow, Russia; 4 Faculty of Biology, Lomonosov Moscow State University, Moscow, Russia; MSU Institute for Mitoengineering, LLC, Moscow, Russia

**Keywords:** age-dependent susceptibility, hepatectomy, hepatic ischemia-reperfusion injury, pediatric liver surgery, vascular exclusion

## Abstract

**Purpose:**

Hepatic ischemia-reperfusion injury remains an important cause of liver dysfunction after major liver surgery. Whether the immature liver responds to prolonged warm ischemia in the same way as the more mature liver is not well defined. This study assessed age-related differences in liver injury in a rat model of extended hepatectomy with vascular exclusion.

**Methods:**

Male Wistar rats aged 3, 6, 9, and 12 weeks underwent approximately 70% hepatectomy with 60 min of vascular exclusion followed by reperfusion. Survival, serum biochemical markers, semi-quantitative histological injury scores, and hepatic malondialdehyde concentration were assessed. Histological data were analyzed using mixed-effects models with animal identity included as a random effect.

**Results:**

Early mortality was high, and overall survival did not differ significantly between age groups. Postoperative biochemical analysis was limited by the small number of surviving animals and was interpreted descriptively. The cumulative histological injury score showed the most consistent age-related pattern. In the mixed-effects model, cumulative injury was associated with age group, operative stage, and the age-by-stage interaction. The main difference was observed after 60 min of ischemia followed by 30 min of reperfusion, when 3-week-old rats had lower cumulative injury scores than older animals. Individual histological components and MDA values were less consistent and were considered supportive findings.

**Conclusion:**

In this severe model of warm ischemia-reperfusion combined with extended hepatectomy, younger rats showed lower cumulative histological injury scores than more mature animals, particularly during the reperfusion phase. These findings suggest that developmental age may influence hepatic tolerance to ischemic stress, but they should be interpreted as experimental and hypothesis-generating rather than directly applicable to clinical ischemia limits in children.

## Introduction

Hepatic ischemia-reperfusion injury (IRI) remains a major cause of postoperative liver dysfunction and failure after major hepatectomy and liver transplantation. Its clinical relevance continues to grow alongside the increasing number of complex liver resections with vascular reconstruction and transplant procedures, where periods of inflow occlusion or total vascular exclusion are often unavoidable. Among various physical, pharmacological, and surgical strategies, hypothermic perfusion and external liver cooling are the most widely used approaches to attenuate hepatocellular injury by reducing metabolic activity and oxidative stress (1–3).

In clinical practice, the generally accepted safe duration of warm ischemia during continuous inflow occlusion (Pringle maneuver or total vascular exclusion) is approximately 40–60 min, although reported thresholds range from 30 to 120 min depending on the condition of the liver parenchyma and the surgical technique employed (2, 4–7). When a longer reconstructive period is anticipated, selective portal vein cannulation, hypothermic perfusion, and active liver cooling are recommended to prevent clinically significant IRI. Despite these measures, severe IRI still contributes to early allograft dysfunction and posthepatectomy liver failure, underscoring the need to better define patient- and procedure-related factors that determine hepatic tolerance to ischemia.

Patient age has been proposed as one of the key modifiers of hepatocyte susceptibility to warm ischemia. Aging is associated with mitochondrial dysfunction, impaired oxidative phosphorylation, and a more pronounced inflammatory and oxidative stress response, which together may lower the ischemic tolerance of the liver. Experimental data in rodent models comparing young and old adult animals demonstrate more severe hepatocellular injury, higher necrosis scores, and worse survival after warm ischemia in older livers (8–11). For instance, Park et al. (8) showed that after 75 min of warm ischemia, all adult rats died within 24 h, whereas young rats all survived, highlighting a stark age-dependent difference in outcome. However, these pivotal studies focused on differences between young and aged adults and did not specifically address the transition through developmental stages relevant to childhood and puberty.

From a clinical perspective, children undergoing liver resection often possess a relatively larger functional liver remnant and fewer comorbidities than adults, which may translate into greater tolerance to inflow occlusion and warm ischemia (12). Nevertheless, there are no direct clinical data comparing IRI severity between pediatric and adult patients, and to our knowledge, no experimental models have systematically evaluated age-dependent tolerance to warm ischemia across the developmental spectrum from weaning to young adulthood.

From a translational perspective, defining the age-related limits of warm ischemia in an experimental model may help refine the use of vascular exclusion techniques in pediatric liver surgery. A better understanding of how the developing liver responds to warm ischemia may be relevant for experimental modeling and for future studies of vascular exclusion in pediatric liver surgery. This experimental study therefore aimed to compare postoperative survival as well as the biochemical and histological markers of hepatic IRI in rats of different ages subjected to extended hepatectomy under prolonged vascular exclusion. We hypothesized that juvenile rats would show lower histological injury after severe warm ischemia-reperfusion than more mature animals, with a possible transition during early maturation.

## Methods

### Animal model

A prospective, controlled, non-randomized experimental study was conducted. The allocation was determined by age, the primary independent variable, which precluded randomization. The animals were maintained under specific pathogen-free conditions. They were housed at 20–24 °C with a 12-hour light/dark cycle, 30%–70% relative humidity, and *ad libitum* access to sterile food and water. Two rats were housed in each T3 cage (Techniplast, Italy) with wood chip bedding. Each rat was individually marked with an ear tag. The rats were weighed and examined daily to assess their general condition. No formal sample size calculation was performed because this was an exploratory experimental study designed to assess age-related injury patterns across developmental stages.

### Ethical approval

This study was approved by the Institutional Review Board of the Dmitry Rogachev National Medical Research Center of Pediatric Hematology, Oncology and Immunology (protocol no. 3/3-22, April 19, 2022). The study is reported in accordance with the ARRIVE guidelines where applicable.

### Anesthesia and analgesia

Inhalation anesthesia was used throughout. Induction was performed with 2.5 vol% isoflurane in an induction chamber, and anesthesia was maintained with spontaneous breathing of 1–2 vol% isoflurane (carrier: air). The heart rate was monitored continuously, and the body temperature was maintained at 37 °C using a multiparameter monitor (Harvard Apparatus, USA). For antibiotic prophylaxis, cefuroxime (16 mg/kg) was administered subcutaneously 30 min before incision. Prior to surgery, a tail vein blood sample was collected to measure baseline serum levels of total and direct bilirubin, alanine aminotransferase (ALT), aspartate aminotransferase (AST), and lactate dehydrogenase (LDH). Postoperative analgesia was provided with ketoprofen (5 mg/kg subcutaneously) before skin closure and then once daily for the first three postoperative days.

### Operative technique

After shaving the abdomen, a bilateral subcostal laparotomy was performed and the liver was mobilized by incising the falciform ligament. Vascular exclusion was achieved by clamping the Glissonean pedicles of the left median and left lateral lobes with bulldog forceps, while the pedicles of the right median and right lateral lobes were ligated with silk 6–0 sutures (Figure 1A). The caudate lobe was not isolated to prevent severe hypotension and intestinal congestion.

**Figure 1:**
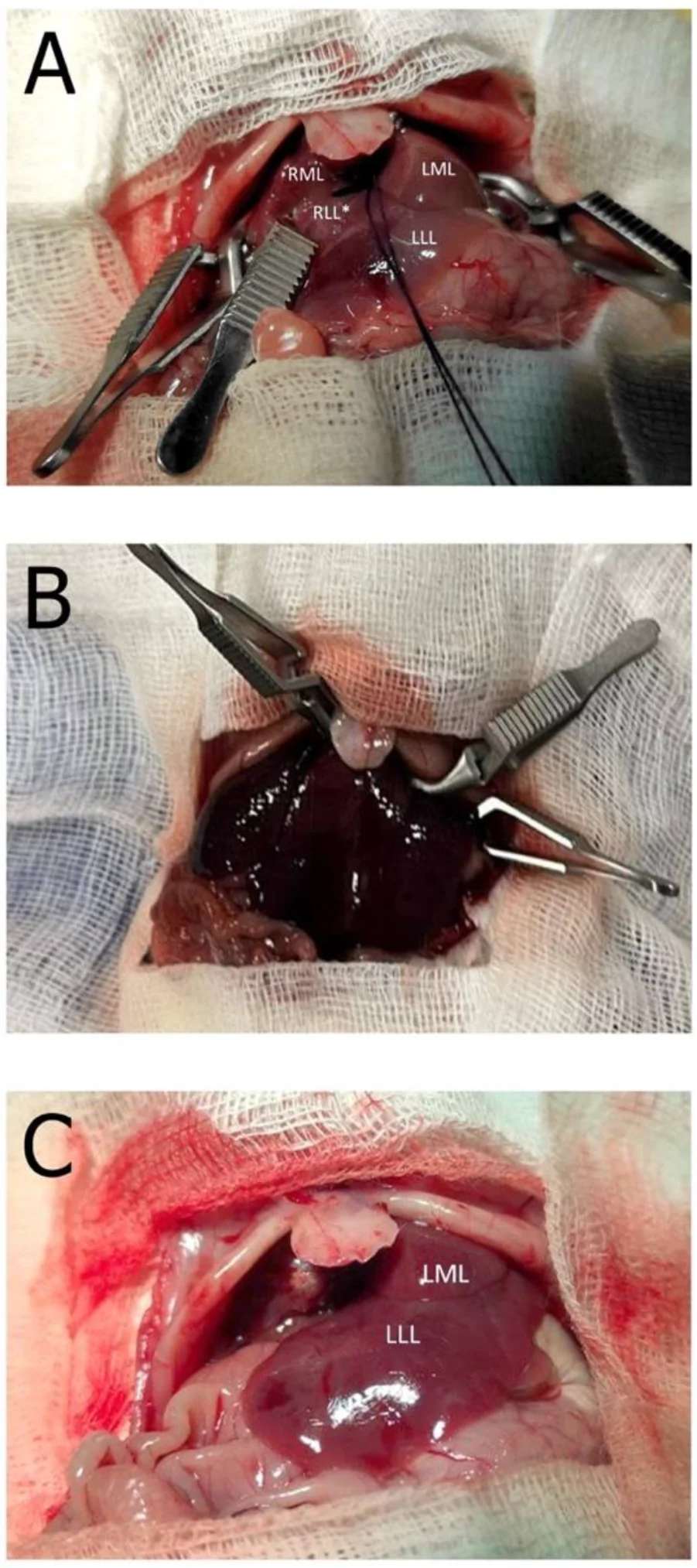
Surgical procedure for extended hepatectomy under vascular exclusion. **(A)** Vascular exclusion: the left median and left lateral lobe pedicles are clamped (bulldog forceps), and the right median and right lateral lobe pedicles are ligated (silk 6-0). **(B)** Right lateral lobectomy performed 30 min after the onset of ischemia (time point I30). **(C)** Reperfusion of the left lobes 60 min after ischemia (time point I60). Anatomical abbreviations: LLL, left lateral lobe; LML, left median lobe; RLL, right lateral lobe; RML, right median lobe.

An atypical biopsy sample from the right median lobe was obtained immediately after vascular isolation (time point I0; Figure 1A). A right lateral lobectomy was then performed sharply, directly above the ligature, 30 min after the onset of ischemia (time point I30; Figure 1B). In the same manner, a right median lobectomy was performed. The clamps were removed from the left lobes after 60 min of ischemia (time point I60; Figure 1C), followed by a 30-minute reperfusion period. After reperfusion, the left median pedicle was ligated, and a left median lobectomy was performed (time point I60 + R30). This sequence resulted in the removal of approximately 70% of the total liver volume under subtotal vascular exclusion, adapting commonly used rat partial hepatectomy approaches (13). Finally, 1-2 mL of sterile saline was instilled into the abdominal cavity, and the wound was closed in layers using Polyglactin 3-0–5-0 sutures.

Upon excision, a ≈25 mg sample of liver tissue was collected for malondialdehyde (MDA) measurement, and the remainder of the lobe was fixed in 10% buffered (pH = 7.0) formalin for histological examination.

### Postoperative period

Postoperative blood samples were obtained on days 3 and 7 and processed as described above. Animals that died before a scheduled sampling point were not included in the corresponding biochemical analysis, and missing values were not replaced or imputed. Surviving animals were euthanized on day 7 under isoflurane anesthesia by sterno-laparotomy followed by cardiac blood collection. Earlier euthanasia was planned if an animal reached humane endpoint criteria, including body weight loss of 20% or more, loss of posture, or severe deterioration in general condition. At necropsy, the liver remnant was collected for histological examination and biochemical analysis.

### Histological examination

Liver samples were fixed, embedded in paraffin, sectioned, and stained with hematoxylin and eosin (H&E) and periodic acid-Schiff (PAS). Sections were examined by light microscopy. Hepatocellular adipose degeneration, parenchymal neutrophilic infiltration, neutrophilic infiltration of injured or fibrotic areas, apoptosis, and ballooning degeneration were graded semi-quantitatively on a 0–3 scale: 0, absent; 1, mild or focal changes; 2, moderate changes; and 3, marked changes. For focal lesions, the number of foci per high-power field (HPF, ×400) was taken into account where applicable (14, 15). A cumulative histological injury score was calculated as the sum of the individual component scores. This cumulative score was used as the summary measure of histological injury. Individual components were analyzed separately. All slides were assessed by a single experienced pathologist who was unaware of the animal age group and sampling time point.

### Assessment of malondialdehyde concentration

Liver tissue for MDA analysis was collected from the resected specimens at each operative time point (I0, I30, I60, and I60 + R30) and from the liver remnant at POD7 or earlier euthanasia, when applicable. Approximately 20–25 mg of liver parenchyma was homogenized in 500 µL of modified RIPA buffer containing 150 mM NaCl, 50 mM Tris-HCl, and 1% NP-40, pH 7.0. The homogenate was centrifuged at 10,000 g for 10 min at 4 °C. The supernatant was divided into 100-µL aliquots and stored at ≤ −68 °C for no longer than 1 month. MDA concentration was measured using a commercial colorimetric kit based on the thiobarbituric acid method (Malondialdehyde Colorimetric Assay Kit; Elabscience, China), according to the manufacturer's protocol. Briefly, 10 µL of homogenate was mixed with 10 µL of clarifying reagent, 150 µL of thiobarbituric acid solution, and 50 µL of 50% acetic acid. The mixture was incubated for 60 min at 95 °C, cooled, and centrifuged for 10 min at 3,100 g at room temperature. Absorbance was measured at 532 nm using a Feyond A400 multimode plate reader (Allsheng, China). MDA values were normalized to total protein content and expressed as nmol/mg protein.

Total protein concentration was measured with the Pierce BCA Protein Assay Kit (Thermo Fisher Scientific, USA). For this assay, 10 µL of sample was mixed with 200 µL of working reagent, incubated for 30 min at 37 °C, and measured at 562 nm using the same plate reader.

### Statistical analysis

Data were summarized descriptively before formal analysis. Continuous variables are reported as mean ± standard deviation or as median with interquartile range, depending on the distribution and the number of observations. Categorical variables are presented as counts and percentages. Postoperative biochemical measurements were analyzed only when samples were available; missing values caused by early death were not imputed. Survival was estimated using Kaplan–Meier curves and compared between age groups with the log-rank test. Fisher's exact test was used for categorical comparisons.

Histological data were analyzed with linear mixed-effects models because several liver samples were obtained from the same animal at different operative stages. Animal identity was entered as a random effect. Age group, operative stage, and the interaction between age group and stage were entered as fixed effects. The cumulative histological injury score was the main histological outcome. Individual histological components were analyzed separately. Pairwise comparisons were performed from estimated marginal means. *P*-values from *post hoc* comparisons were adjusted using the Benjamini-Hochberg false discovery rate procedure. Since the histological scores were semi-quantitative ordinal variables, ordinal mixed-effects models were also tested as sensitivity analyses. When these models produced unstable estimates because of sparse categories, excess zero values, or separation, the results were not used for inference and the corresponding variables were described qualitatively.

MDA concentrations were analyzed with mixed-effects models using the same structure: animal identity as a random effect, and age group, operative stage, and their interaction as fixed effects. Serum biochemical markers were analyzed descriptively and interpreted with caution because few animals survived to the later postoperative sampling points.

Mixed-effects models were fitted in R using the lme4, lmerTest, emmeans, and ordinal packages. Graphs were prepared in GraphPad Prism. A two-sided *p*-value below 0.05 was considered statistically significant. Additional mixed-effects model results, FDR-adjusted pairwise comparisons, and score distributions are provided in Supplementary Figure S1 and Supplementary Tables S1–S4.

## Results

A total of 27 male Wistar rats were assigned to four groups based on their age at surgery (Table 1): 3 weeks (*n* = 7), 6 weeks (*n* = 7), 9 weeks (*n* = 8), and 12 weeks (*n* = 5).

**Table 1:** Distribution of animals by age.

Age (weeks)	Developmental stage	Number of animals
3	Weaning from breastfeeding	7
6	Prepubertal age	7
9	Puberty	8
12	Young adult	5
The developmental stages are approximate and based on standard rat developmental biology (16).		

### Animal survival

No intraoperative technical complications were recorded. Six rats died during vascular exclusion: two between I30 and I60, and four during the reperfusion period. A further 15 animals died within the first postoperative day. Overall survival did not differ significantly between age groups on Kaplan–Meier analysis with the log-rank test (*p* = 0.247; Figure 2A).

**Figure 2:**
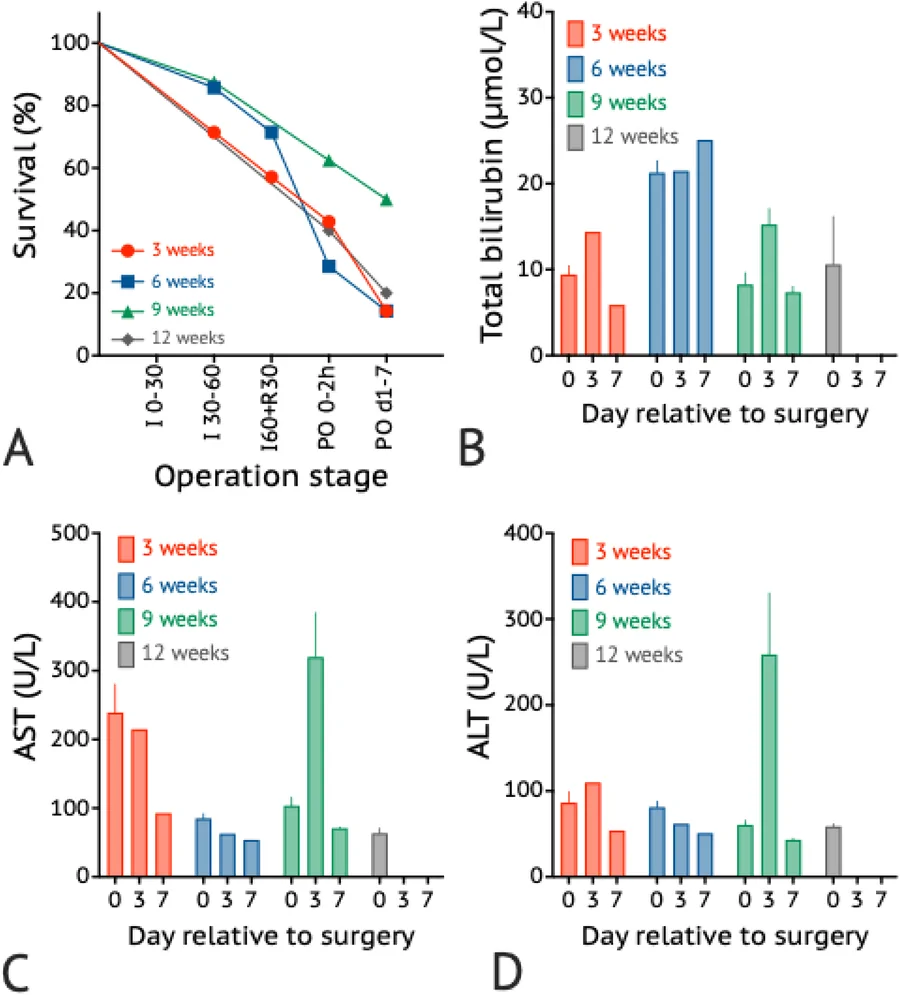
Postoperative survival and serum markers of hepatocellular injury. **(A)** Survival across intraoperative and early postoperative stages in 3-, 6-, 9-, and 12-week-old rats undergoing extended hepatectomy under vascular exclusion. **(B–D)** Serial changes in serum total bilirubin, ALT, and AST at baseline and on postoperative days 3 and 7. Biochemical data are shown descriptively because the number of animals available at later postoperative time points was limited by high early mortality. Data are presented as mean ± SEM.

**Figure 3:**
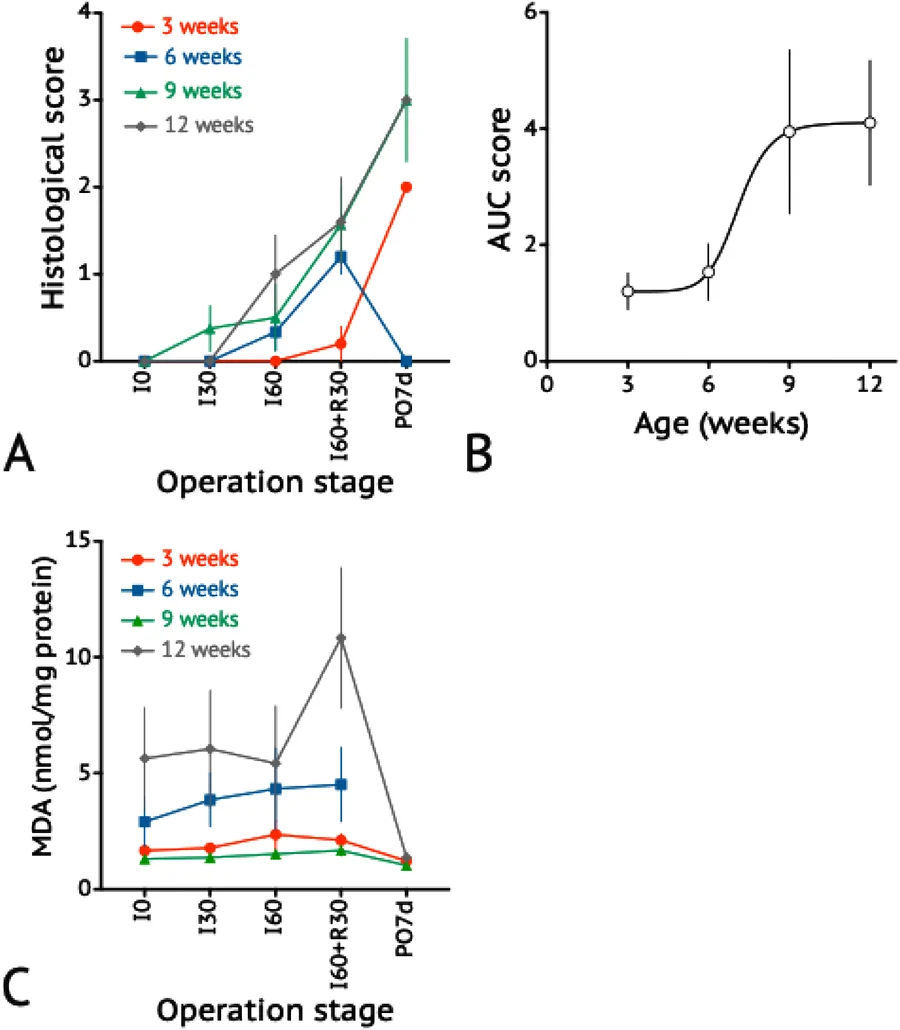
Cumulative histological injury and hepatic malondialdehyde concentration**. (A)** Mean cumulative histological injury score at each operative stage and on POD7. Cumulative injury increased during prolonged ischemia and reperfusion, with the lowest scores observed in 3-week-old animals at I60 + R30. **(B)** Descriptive area under the curve of the cumulative histological score across sampling points by age group. **(C)** Hepatic MDA concentration at each operative stage and on POD7. MDA values showed inter-individual variability and no clear age-specific pattern. Data are presented as mean ± SEM.

### Biochemical markers of hepatocellular injury

Postoperative biochemical analysis was limited by the high early mortality. Available samples showed the expected postoperative changes in liver injury markers, with the most pronounced deviations from baseline observed on POD3 for ALT, AST, and total bilirubin (Figures 2B–D). However, because only a small number of animals survived to the later sampling points, age-stratified biochemical comparisons were not considered reliable as primary evidence. These results were therefore interpreted descriptively and used only as supportive indicators of postoperative liver injury.

### Histological markers of hepatocellular injury

Histological changes were assessed at the intraoperative time points I0, I30, I60, and I60 + R30, with additional terminal postoperative samples available only in a small number of animals because of early mortality. The cumulative histological injury score showed the clearest age-related pattern among the assessed endpoints (Figure 3A). In the linear mixed-effects model, with animal identity included as a random effect, the integrated histological score was significantly associated with age group (*p* = 0.0016), operative stage (*p* < 0.0001), and the interaction between age group and operative stage (*p* < 0.0001). The model including the interaction term provided a better fit than the model without interaction (*p* < 0.0001), indicating that the course of histological injury differed between age groups rather than increasing uniformly over time. At the early time points, cumulative scores were low or absent in most animals. Injury became more apparent after 60 min of ischemia and was most pronounced after reperfusion. At I60 + R30, 3-week-old rats had significantly lower cumulative injury scores than 6-week-old (*p* = 0.0229), 9-week-old (*p* = 0.0024), and 12-week-old animals (*p* = 0.0024) after false discovery rate correction. Scores in the 6-, 9-, and 12-week groups were more similar to each other at this stage.

Within-group comparisons also showed different injury trajectories. In 9- and 12-week-old rats, cumulative scores increased markedly from the early ischemic stages to I60 + R30 and terminal postoperative sampling. In contrast, the increase was less pronounced in the youngest animals. Postoperative histological findings, especially those from day 7, were interpreted cautiously because of the small number of surviving animals. Individual histological components were less consistent than the cumulative score. Some components were sparse or dominated by zero values and were therefore treated as supportive descriptive findings. The cumulative score was retained as the main histological endpoint.

### Hepatic malondialdehyde concentration

Hepatic MDA concentrations showed considerable inter-individual variability. In the mixed-effects analysis, MDA did not show a clear age-dependent pattern. Values tended to follow the expected ischemia-reperfusion course, with higher levels during prolonged ischemia and early reperfusion and lower values in the late postoperative samples, but this pattern was variable. MDA was therefore considered a supportive marker of tissue lipid peroxidation rather than a primary endpoint for age-related differences.

Overall, the histological data indicated that liver injury accumulated during prolonged ischemia and reperfusion, but the magnitude of this accumulation depended on developmental age. The most consistent difference was observed during the reperfusion phase, where 3-week-old animals showed lower cumulative histological injury than older groups.

## Discussion

This study examined whether developmental age modifies hepatic tolerance to severe warm ischemia-reperfusion in a rat model of extended hepatectomy. The model was deliberately stressful: 70% liver resection combined with 60 min of vascular exclusion and subsequent reperfusion. As expected, this resulted in high early mortality and left only limited postoperative biochemical material for analysis. Survival did not differ significantly between age groups. The main finding, therefore, was not survival-based, but histological: cumulative liver injury increased with operative stage, and the magnitude of this increase depended on animal age.

Among the available endpoints, the cumulative histological injury score carried most of the signal. In the mixed-effects analysis, the score was associated with age group, operative stage, and the age-by-stage interaction. This was essential for the present design, in which several liver samples came from the same animal. The most consistent difference was seen after 60 min of ischemia followed by 30 min of reperfusion, where 3-week-old rats had lower cumulative injury scores than 6-, 9-, and 12-week-old animals. In other words, the youngest animals did not simply have lower baseline injury; they showed a different response during the phase in which ischemic damage became histologically apparent. Individual histological components were less stable than the cumulative score. Some findings, such as ballooning degeneration and apoptosis, were rare. Parenchymal neutrophilic infiltration showed a stage-related pattern, but positive observations were still sparse. Fibrous-area infiltration was largely a late postoperative finding and was limited by the very small number of animals surviving to later time points. For this reason, these components should be read as supportive details rather than separate mechanistic endpoints. The cumulative score is a more appropriate summary of the tissue response in this model.

The MDA data should also be interpreted cautiously. MDA concentrations followed a biologically plausible ischemia-reperfusion pattern, with higher values during prolonged ischemia and early reperfusion and lower values in late postoperative samples. However, the signal was variable and did not provide a robust age-specific effect. Thiobarbituric acid-based MDA assays are useful as accessible indicators of lipid peroxidation, but they are not specific markers of oxidative injury and may be affected by sample handling and post-sampling reactions (17). In the present study, MDA was therefore treated as a supportive biochemical readout, not as proof of an age-dependent oxidative mechanism. A more complete analysis would require additional markers such as 4-HNE, 8-OHdG, glutathione status, and antioxidant enzyme activity.

The estimated transition in susceptibility occurred around the peripubertal period. The proximity of this transition to the peripubertal period is noteworthy, although the present study cannot explain it mechanistically. Puberty in rats is accompanied by endocrine and metabolic remodeling, and sex steroids may influence hepatic inflammation, mitochondrial function, and ischemia-reperfusion pathways (16, 18–23). Still, the present experiment was not designed to test this mechanism. Hormone concentrations were not measured, receptor expression was not assessed, and only male animals were included. Thus, puberty-related endocrine changes remain a possible explanation rather than a conclusion. Dedicated studies including male and female animals, hormone profiling, and receptor-level analysis would be needed to address this question directly. Our findings are consistent with earlier experimental work showing greater hepatic vulnerability to ischemia-reperfusion in older animals (8–11). Those studies mainly compared young adult and aged rodents. The present model differs in that it focuses on earlier developmental stages, from weaning to young adulthood. The results suggest that the shift in susceptibility may begin much earlier than senescence, during the transition from juvenile to more mature physiology. This does not mean that rat age groups can be directly mapped onto pediatric surgical age categories. It does suggest, however, that developmental age is not a neutral variable in experimental models of hepatic ischemia-reperfusion.

The clinical interpretation should remain cautious. Children undergoing major liver resection often have favorable functional reserve, and clinical series show that complex pediatric hepatectomies, including procedures with small future liver remnants and vascular exclusion, can be performed safely in specialized centers (12, 24–27). These observations are in line with the idea that the pediatric liver may differ from the adult liver in its response to surgical stress. However, our data do not define safe ischemia times for children and should not be used to justify longer vascular exclusion in clinical practice. Rather, they support considering developmental age in future experimental and clinical studies of hepatic ischemia tolerance. This cautious interpretation is consistent with the principles of evidence-based decision-making in pediatric hematology and oncology (28).

These findings should be read with several limitations in mind. First, the high early mortality made survival and postoperative biochemical endpoints underpowered. Day 7 histology was also based on very few animals and may be affected by survival bias. Second, the histological scores were semi-quantitative and ordinal. Although mixed-effects models were used to account for repeated sampling within animals, ordinal mixed models were unstable for several variables because of sparse categories and excess zero values. Third, oxidative stress was assessed using MDA alone. Finally, the study included only male rats and did not examine endocrine or molecular mechanisms. Despite these limitations, the mixed-effects reanalysis supports the main observation: in this severe model of warm ischemia-reperfusion combined with extended hepatectomy, younger rats showed lower cumulative histological injury than more mature animals, particularly during the reperfusion phase. The results should be regarded as hypothesis-generating rather than directly prescriptive for pediatric surgery. They indicate that developmental age may influence hepatic tolerance to ischemic stress and should be incorporated into future preclinical models of liver resection and vascular exclusion.

## Data Availability

The original contributions presented in the study are included in the article/Supplementary Material, further inquiries can be directed to the corresponding author.
