# Supplementary material for: Age-related hepatic tolerance to ischemia-reperfusion injury in a rat model of extended hepatectomy
Source: Front Surg. 2026 Oct 9;13:1902759. doi: 10.3389/fsurg.2026.1902759 (PMC13693952; doi:10.3389/fsurg.2026.1902759)
Supplement: Supplementary file 1 [file Supplementaryfile1.pdf]

Supplementary material

Age-related hepatic tolerance to ischemia-reperfusion injury in a rat model of extended hepatectomy

Supplementary statistical analyses are presented for the cumulative histological injury score and for selected secondary histological components. The main mixed-effects model used animal identity as a random effect and age group, operative stage, and their interaction as fixed effects.

Supplementary Figure S1

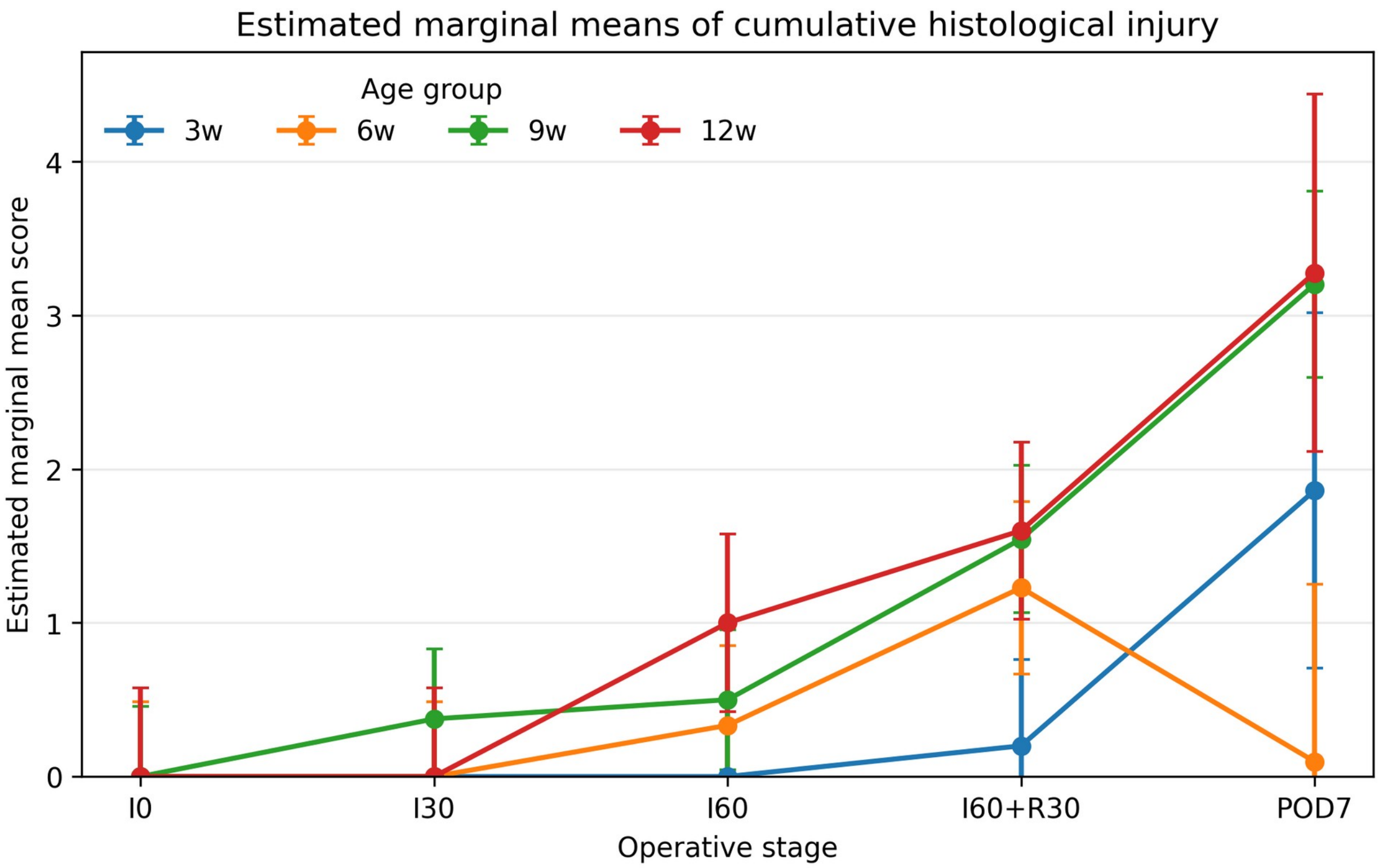

Supplementary Figure S1. Estimated marginal means of the cumulative histological injury score derived from the linear mixed-effects model. Animal identity was included as a random effect; age group, operative stage, and their interaction were included as fixed effects. Error bars indicate 95% confidence intervals.

Supplementary Table S1. Linear mixed-effects model for the cumulative histological injury score

Fixed effect	NumDF	DenDF	F value	p value
Age group	3	37.220	6.2123	0.001575
Operative stage	4	84.847	33.9228	<0.0001
Age group × stage	12	84.787	4.2926	2.746×10^{-5}

Note. Type III analysis of variance with Satterthwaite degrees of freedom. The model including the interaction term fitted the data better than the model without interaction (likelihood-ratio test, $p = 6.676 \times 10^{-5}$).

Supplementary Table S2. FDR-adjusted age-group comparisons at I60+R30

Comparison	Estimate	SE	df	t ratio	FDR-adjusted p value
3w − 6w	−1.0280	0.400	111.6	−2.571	0.0229
3w − 9w	−1.3452	0.372	106.9	−3.613	0.0024
3w − 12w	−1.4000	0.405	101.5	−3.455	0.0024
6w − 9w	−0.3172	0.372	106.9	−0.852	0.4754
6w − 12w	−0.3720	0.405	101.5	−0.918	0.4754
9w − 12w	−0.0548	0.378	95.3	−0.145	0.8851

Note. Contrasts were obtained from estimated marginal means of the linear mixed-effects model and adjusted by the Benjamini-Hochberg false discovery rate procedure.

Supplementary Table S3. Distribution of histological score categories

Outcome	0	1	2	3	5	Total
Cumulative histological injury score	77	14	10	6	1	108
Hepatocellular adipose degeneration	84	22	2	0	0	108
Parenchymal neutrophilic infiltration	98	9	1	0	0	108
Neutrophilic infiltration of injured/fibrotic areas	102	4	2	0	0	108
Apoptosis	100	8	0	0	0	108
Ballooning degeneration	104	4	0	0	0	108

Note. The table shows the number of observations in each score category. The cumulative score could exceed 3 because it was calculated as the sum of individual component scores.

Supplementary Table S4. Secondary mixed-effects analyses for individual histological components

Outcome	Age group p value	Stage p value	Age group × stage p value	Interpretation
Parenchymal neutrophilic infiltration	0.06822	0.01115	0.05128	Stage-dependent pattern; age and interaction showed trends.
Neutrophilic infiltration of injured/fibrotic areas	<0.0001	<0.0001	<0.0001	Predominantly late postoperative finding; interpreted with caution.
Apoptosis	0.05590	0.01852	0.03435	Rare positive observations; supportive component.
Ballooning degeneration	0.5775	0.2579	0.5390	Rare and not significantly associated with age or stage.

Note. Linear mixed-effects models used the same structure as the main histological model. Because individual component scores were sparse and ordinal, these results were treated as secondary supportive findings rather than as independent mechanistic endpoints.
