# Supplementary material for: Age-related hepatic tolerance to ischemia-reperfusion injury in a rat model of extended hepatectomy
Source: Front Surg. 2026 Oct 9;13:1902759. doi: 10.3389/fsurg.2026.1902759 (PMC13693952; doi:10.3389/fsurg.2026.1902759)
Supplement: Supplementary file 2 [file Table1.docx]

*Age-related hepatic tolerance to ischemia-reperfusion injury in a rat model of extended hepatectomy*

Supplementary statistical analyses are presented for the cumulative histological injury score and for selected secondary histological components. The main mixed-effects model used animal identity as a random effect and age group, operative stage, and their interaction as fixed effects.

**Supplementary Figure S1.** Estimated marginal means of the cumulative histological injury score derived from the linear mixed-effects model. Animal identity was included as a random effect; age group, operative stage, and their interaction were included as fixed effects. Error bars indicate 95% confidence intervals.

# Supplementary Table S1. Linear mixed-effects model for the cumulative histological injury score

| **Fixed effect** | **NumDF** | **DenDF** | **F value** | **p value** |
| --- | --- | --- | --- | --- |
| Age group | 3 | 37.220 | 6.2123 | 0.001575 |
| Operative stage | 4 | 84.847 | 33.9228 | <0.0001 |
| Age group × stage | 12 | 84.787 | 4.2926 | 2.746 × 10⁻5 |

**Note.** Type III analysis of variance with Satterthwaite degrees of freedom. The model including the interaction term fitted the data better than the model without interaction (likelihood-ratio test, p = 6.676 × 10⁻5).

Supplementary Table S2. FDR-adjusted age-group comparisons at I60+R30

| **Comparison** | **Estimate** | **SE** | **df** | **t ratio** | **FDR-adjusted p value** |
| --- | --- | --- | --- | --- | --- |
| 3w − 6w | −1.0280 | 0.400 | 111.6 | −2.571 | 0.0229 |
| 3w − 9w | −1.3452 | 0.372 | 106.9 | −3.613 | 0.0024 |
| 3w − 12w | −1.4000 | 0.405 | 101.5 | −3.455 | 0.0024 |
| 6w − 9w | −0.3172 | 0.372 | 106.9 | −0.852 | 0.4754 |
| 6w − 12w | −0.3720 | 0.405 | 101.5 | −0.918 | 0.4754 |
| 9w − 12w | −0.0548 | 0.378 | 95.3 | −0.145 | 0.8851 |

**Note.** Contrasts were obtained from estimated marginal means of the linear mixed-effects model and adjusted by the Benjamini-Hochberg false discovery rate procedure.

# Supplementary Table S3. Distribution of histological score categories

| **Outcome** | **0** | **1** | **2** | **3** | **5** | **Total** |
| --- | --- | --- | --- | --- | --- | --- |
| Cumulative histological injury score | 77 | 14 | 10 | 6 | 1 | 108 |
| Hepatocellular adipose degeneration | 84 | 22 | 2 | 0 | 0 | 108 |
| Parenchymal neutrophilic infiltration | 98 | 9 | 1 | 0 | 0 | 108 |
| Neutrophilic infiltration of injured/fibrotic areas | 102 | 4 | 2 | 0 | 0 | 108 |
| Apoptosis | 100 | 8 | 0 | 0 | 0 | 108 |
| Ballooning degeneration | 104 | 4 | 0 | 0 | 0 | 108 |

**Note.** The table shows the number of observations in each score category. The cumulative score could exceed 3 because it was calculated as the sum of individual component scores.

# Supplementary Table S4. Secondary mixed-effects analyses for individual histological components

| **Outcome** | **Age group p value** | **Stage p value** | **Age group × stage p value** | **Interpretation** |
| --- | --- | --- | --- | --- |
| Parenchymal neutrophilic infiltration | 0.06822 | 0.01115 | 0.05128 | Stage-dependent pattern; age and interaction showed trends. |
| Neutrophilic infiltration of injured/fibrotic areas | <0.0001 | <0.0001 | <0.0001 | Predominantly late postoperative finding; interpreted with caution. |
| Apoptosis | 0.05590 | 0.01852 | 0.03435 | Rare positive observations; supportive component. |
| Ballooning degeneration | 0.5775 | 0.2579 | 0.5390 | Rare and not significantly associated with age or stage. |

**Note.** Linear mixed-effects models used the same structure as the main histological model. Because individual component scores were sparse and ordinal, these results were treated as secondary supportive findings rather than as independent mechanistic endpoints.
